# Fanconi Anemia in Mexican Patients: Molecular Spectrum and Clinical Manifestations in a Case Series

**DOI:** 10.3390/ijms27094015

**Published:** 2026-04-30

**Authors:** Fernando Alexis Flores-Leura, Sinhue Alejandro Brukman-Jiménez, Alfredo Corona-Rivera, Idalid Cuero-Quezada, José de Jesús Pérez-Becerra, Juan Antonio Ramírez-Corona, Víctor Ulises Rodríguez-Machuca, María Magdalena Ortiz-Sandoval, Fátima Jazmín Hinojosa-Piña, Olga Lidia Navarro-Barba, Jorge Román Corona-Rivera, Lucina Bobadilla-Morales

**Affiliations:** 1 Human Genetics PhD Program, Department of Molecular Biology and Genomics, Centro Universitario de Ciencias de la Salud, Universidad de Guadalajara, Guadalajara 44340, Mexico; fernando.flores8452@alumnos.udg.mx (F.A.F.-L.); idalidcuero95@gmail.com (I.C.-Q.); perezbecerra95@gmail.com (J.d.J.P.-B.); ju.an.ram.cor@gmail.com (J.A.R.-C.); vu.rodriguez11@gmail.com (V.U.R.-M.); 2 Human Genetics Institute “Dr. Enrique Corona Rivera”, Department of Molecular Biology and Genomics, Centro Universitario de Ciencias de la Salud, Universidad de Guadalajara, Guadalajara 44340, Mexico; alfredo.corona@academicos.udg.mx (A.C.-R.); roman.corona@academicos.udg.mx (J.R.C.-R.); 3 Cytogenetics Unit, Hospital Civil de Guadalajara Dr. Juan I. Menchaca, Guadalajara 44340, Mexico; brukman.alejandro@gmail.com; 4 Transplant Unit of Henatopoietic Progenitor, Pediatric Hematology and Oncology Department, Hospital Civil de Guadalajara Dr. Juan I. Menchaca, Guadalajara 44340, Mexico; drammros2@gmail.com; 5 Centro Universitario de Ciencias de la Salud, Universidad de Guadalajara, Guadalajara 44340, Mexico; fatima.hinojosa4561@alumnos.udg.mx; 6 Division of Auxiliary Diagnostic and Treatment Services, Hospital Civil de Guadalajara Dr. Juan I. Menchaca, Guadalajara 44340, Mexico; onavarro@hcg.gob.mx

**Keywords:** Fanconi anemia, Mexico, *FANCA*, *FANCC*, *FANCE*, case series

## Abstract

Fanconi anemia (FA) is a rare inherited disorder characterized by genomic instability, congenital anomalies, and progressive bone marrow failure; such manifestations may vary across populations, partly due to differences in genetic background. This study aims to describe the clinical and molecular spectrum of FA in Mexican patients. A total of 14 patients with clinical suspicion of FA were evaluated; cytogenetic and molecular analyses were successfully performed using MLPA and NGS. Clinically, short stature was present in 100% (n = 14) of the patients, followed by upper limb abnormalities, which were present in 78.6% (n = 11) of the patients, and microphthalmia, which was present in 71.4% (n = 10) of the patients. Molecular analysis identified pathogenic variants in *FANCA* (78.6%, n = 11), *FANCC* (14.3%, n = 2), and *FANCE* (7.1%, n = 1), with a relatively balanced distribution of homozygous (57.1%, n = 8) and compound heterozygous variants (42.9%, n = 6). Notably, the *FANCA*:c.3931_3932del variant was recurrent in six patients from the same geographic region (Michoacan), suggesting possible regional enrichment. Our findings expand the clinical and molecular characterization of FA in Mexican patients and underscore the importance of integrating phenotypic and genomic data to better understand population-specific patterns of this disorder.

## 1. Introduction

Fanconi anemia (FA) is the most common inherited bone marrow failure syndrome, with an estimated global incidence of approximately 1 in 100,000–160,000 live births [1]. It is characterized by chromosomal instability, hematologic abnormalities, and a marked predisposition to the early development of malignancies such as chronic myeloid leukemia. FA presents a broad and heterogeneous clinical spectrum affecting multiple organ systems. The most frequent developmental anomalies include short stature, cutaneous pigmentary changes, radial ray malformations, microcephaly, and renal abnormalities. Notably, the phenotype is highly variable, even among patients with similar genetic backgrounds, and may be grouped into clinical associations such as VACTERL-H and PHENOS, which aid in guiding diagnostic suspicion [2,3,4,5].

The disease arises from mutations in genes involved in DNA repair through the FA/BRCA pathway. This pathway represents a complex network responsible for recognizing and repairing interstrand DNA crosslinks (ICLs). Disruption of this system leads to the accumulation of DNA damage, resulting in increased chromosomal breaks and genomic instability. To date, at least 23 genes have been identified, collectively known as FANC genes, including *FANCA*, *FANCB*/*FAAP95*, *FANCC*, *FANCD1*/*BRCA2*, *FANCD2*, *FANCE*, *FANCF*, *FANCG*/*XRCC9*, *FANCI*, *FANCJ*/*BRIP1*, *FANCL*, *FANCM*, *FANCN*/*PALB2*, *FANCO*/*RAD51C*, *FANCP*/*SLX4*, *FANCQ*/*ERCC4*, *FANCR*/*RAD51*, *FANCS*/*BRCA1*, *FANCT*/*UBE2T*, *FANCU*/*XRCC2*, *FANCV*/*REV7*, *FANCW*/*RFWD3*, and *FANCX*/*FAP100*. Most of these genes follow an autosomal recessive inheritance pattern; however, exceptions exist, such as *FANCB* (X-linked) and *FANCR*/*RAD51* (autosomal dominant) [6,7].

Among FANC genes, *FANCA* is the most frequently affected, accounting for approximately 64% of cases. Variants in other genes contribute to a smaller proportion, with *FANCC* and *FANCG* representing the next most commonly affected genes, collectively accounting for approximately 20% of cases. The initial diagnostic approach is based on clinical suspicion, followed by cytogenetic assays used to assess chromosomal instability induced by DNA crosslinking agents such as diepoxybutane (DEB) or mitomycin C (MMC). Diagnostic confirmation is subsequently achieved through molecular testing aimed to identify pathogenic variants in FA-associated genes. The implementation of next-generation sequencing (NGS), together with copy number variation (CNV) analysis using multiplex ligation-dependent probe amplification (MLPA), has significantly improved diagnostic accuracy, with reported diagnostic yields approaching 90%, enabling the detection of both single-nucleotide variants and structural alterations [1].

Although the molecular basis of Fanconi anemia has been extensively characterized in diverse populations, data regarding its clinical and genetic spectrum in Latin American populations remain limited. In this context, the study of underrepresented populations is essential to generate relevant evidence that may improve diagnostic strategies and expand current knowledge of the disease. Furthermore, population-specific genetic variation, including recurrent or founder variants, has been described in certain FA genes, highlighting the influence of demographic and historical factors on the mutational spectrum of the disease.

In this study, we describe the clinical manifestations and molecular findings in a cohort of Mexican patients with Fanconi anemia. By integrating cytogenetic testing, CNV analysis by MLPA, and NGS, we aim to contribute to the characterization of the clinical and molecular landscape of FA in this population and to provide insight into its phenotypic and genetic variability.

## 2. Results

A total of 14 patients with clinical suspicion of Fanconi anemia whose clinical and molecular data were available were included in the analysis. Among them, eight were male and six were female, resulting in a male-to-female ratio of 1.3:1. The age at diagnosis ranged from 3 to 15 years.

Hematological involvement suggestive of bone marrow failure was observed in 11 of the 14 patients, presenting as anemia, thrombocytopenia, or pancytopenia, while three patients did not exhibit these findings at the time of evaluation. The age at onset of hematological manifestations ranged from 4 to 8 years, based on the clinical assessment at the time of examination.

All patients underwent an induced chromosomal instability (CI) test, which revealed increased chromosomal breakage and the presence of radial figures, findings consistent with Fanconi anemia. Conventional karyotype analysis showed no visible chromosomal abnormalities in this case series.

A comprehensive clinical evaluation was performed in all patients using the VACTERL-H and PHENOS clinical criteria, two widely used frameworks for the clinical assessment of individuals with FA. The clinical analysis showed that short stature was present in 100% (n = 14) of patients, followed by upper limb abnormalities in 78.6% (n = 11) and microphthalmia in 71.4% (n = 10) of the patients. No V, T, E, H, N, or O features were identified in our study based on the available clinical and imaging evaluations. A detailed summary of the clinical findings observed in the study is presented in Table 1.

**Figure 1:**
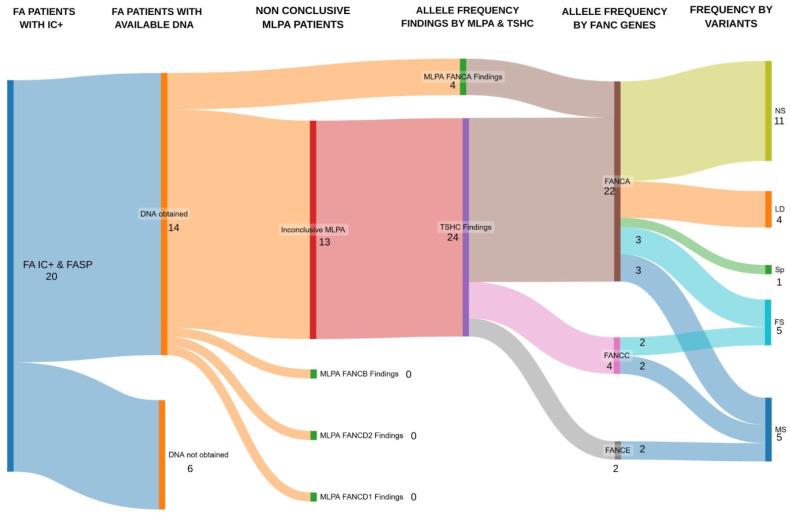
Diagram illustrating the cytogenomic diagnostic workflow applied for patients with suspected Fanconi anemia in our group of patients, including MLPA and next-generation sequencing, which was used to identify pathogenic variants in FANC genes. FASP: Fanconi anemia suspicious phenotype; IC: Chromosomal instability test; MLPA: Multiplex ligation-dependent probe amplification; TSHC: TrueSight hereditary cancer panel; FA: Fanconi anemia. NS: nonsense; LD: large deletion; Sp: splicing; FS: frameshift; MS: missense. Made using the website SankeyMATIC.com (https://sankeymatic.com/build/ accessed on 17 April 2026).

MLPA analysis identified three patients with copy number alterations in *FANCA*. One patient harbored a novel homozygous deletion encompassing seven exons (exons 12–18), which has not been previously reported in the literature. In addition, two patients showed heterozygous deletions involving three exons (1–3) and eleven exons (18–28), respectively; both rearrangements have been previously reported in the literature. No CNVs were detected in the remaining patients by this method, and no copy number alterations were observed in *FANCD1*, *FANCD2*, or *FANCB* within this cohort. The overall cytogenomic diagnostic workflow applied in this cohort is illustrated in Figure 1.

**Figure 2:**
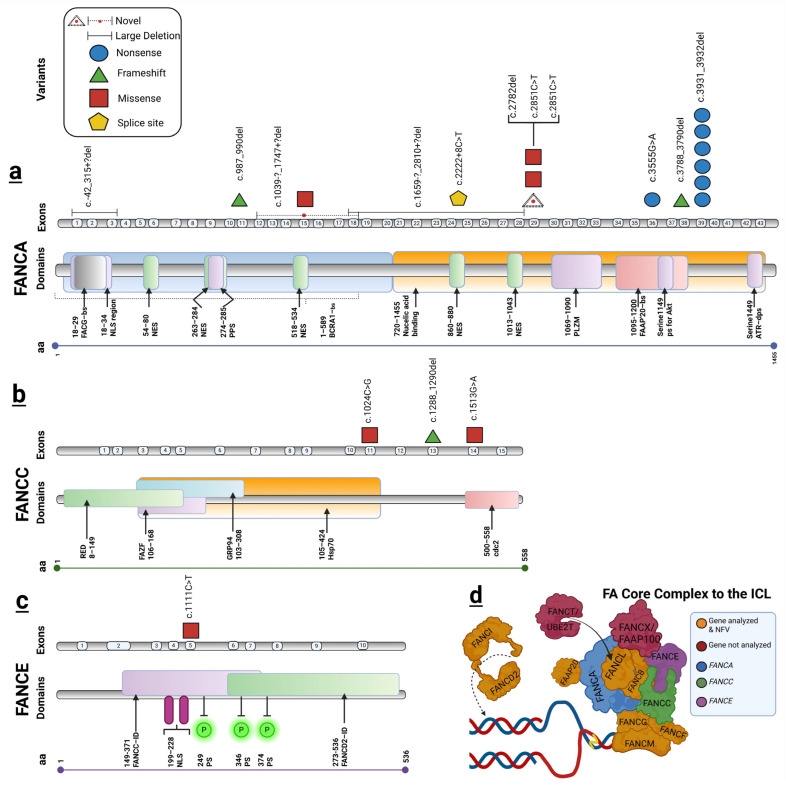
Schematic representation of the distribution of germline variants identified in *FANCA*, *FANCC*, and *FANCE* in the study cohort. Variants are mapped according to their position across gene exons and corresponding protein domains. Different symbols indicate variant types, including nonsense, frameshift, missense, splice-site variants, and large deletions, as shown in the legend. (**a**) *FANCA* spectrum of germline variants. Domains for FANCG-bs (FANCG binding site), NLS region (nuclear localization signal region), NES (nuclear export signals), PPS (putative peroxidase site), PLZM (partial leucine zipper motif), FAAP20-bs (FAAP binding site), 1149 serine-ps (phosphorylation site) for Akt, and 1449 serine ATR-dps (dependent phosphorylation site) are indicated. (**b**) *FANCC* spectrum of germline variants found. Domains for RED (NADPH cytochrome-P450 reductase), FAZF, ZBTB32 or TZFP (Fanconi anemia zinc finger), molecular chaperone GRP94, HSP70 (heat shock protein 70), and cdc2 (cyclin-dependent kinase) are indicated. (**c**) *FANCE* variant found: FANCC-ID (*FANCC* interaction domains), FANCD2-ID (*FANCD2* interaction domains), NLS (nuclear localization signal), and P (phosphorylation site) are indicated. (**d**) Schematic representation of Fanconi anemia “core complex” composed by 9 proteins. The genes in that complex in which pathogenic variants were found were as follows: *FANCA* (blue), *FANCC* (green) and *FANCE* (purple); genes analyzed and NFV (not found variant) (orange) and genes not analyzed (red) by TSHC (TruSight hereditary cancer) panel are indicated [11,12,13,14]. Created in BioRender. Brukman, A. (2026) https://BioRender.com/1nwxdav.

NGS was subsequently performed for the 13 patients who remained molecularly unresolved after MLPA analysis using the TruSight hereditary cancer panel. This analysis identified 11 pathogenic or likely pathogenic individual variants across genes associated with the Fanconi anemia pathway. Specifically, seven variants were detected in *FANCA*, three in *FANCC*, and one in *FANCE*. A comprehensive description of the molecular findings, including genomic annotations and predicted effects, is provided in Table 2. Furthermore, a schematic representation of the distribution of the identified variants across each gene is provided in Figure 2.

### 2.1. FANCA Variants

A total of seven distinct sequence-level variants were identified in *FANCA* by NGS, excluding copy number variants detected by MLPA, making it the most frequently altered gene in our patients. These variants comprised three frameshift mutations resulting from small deletions, two nonsense variants, one missense substitution, and one intronic variant predicted to affect splicing. Most of the identified variants have been previously reported in the literature, with the exception of *FANCA*:c.2782del, detected in patient FA1, which represents a novel alteration in our cohort.

Most of the identified *FANCA* variants are predicted to result in loss of function, either through premature truncation or disruption of protein structure. *FANCA* is a central component of the FA core complex, which is required for the monoubiquitination of *FANCD2* and *FANCI*, a critical step in the FA/BRCA DNA repair pathway. Disruption of *FANCA* function impairs this process, leading to defective repair of DNA interstrand crosslinks and increased chromosomal instability. As shown in Figure 2, the identified *FANCA* variants are distributed across multiple exons and functional domains, with several truncating variants affecting regions critical for protein stability and interaction within the FA core complex.

Among these variants, *FANCA*:c.3931_3932del was the most recurrent, being detected in six patients in both heterozygous and homozygous states, including three siblings (FA5, FA6, and FA7) who were previously reported by our group [10]. Interestingly, all individuals carrying this variant originated from Michoacán, Mexico, suggesting possible regional enrichment., although additional studies will be necessary to confirm this observation.

Overall, the combination of MLPA and NGS analyses allowed the identification of the molecular genotype in the majority of the cohort, revealing six patients with homozygous variants and five patients with compound heterozygous alterations affecting *FANCA*.

### 2.2. FANCC Variants

Three variants were identified in *FANCC*, including two missense substitutions and one frameshift mutation resulting from a small deletion. All variants have been previously reported in the literature and are consistent with pathogenic alterations associated with FA.

*FANCC* plays a key role in maintaining the stability and proper assembly of the FA core complex. Pathogenic variants in *FANCC* are known to disrupt protein interactions within the complex, impairing the downstream activation of the FA/BRCA pathway. This functional disruption may contribute to a more pronounced clinical phenotype, particularly in relation to earlier onset of bone marrow failure. As illustrated in Figure 2, *FANCC* variants identified in our cohort are located within regions associated with protein stability and interaction, supporting their potential impact on FA core complex assembly.

Regarding their zygosity, one patient carried the variants in a compound heterozygous state, whereas another patient presented a homozygous variant, in accordance with the autosomal recessive inheritance pattern of the disorder.

### 2.3. FANCE Variant

A single variant was identified on *FANCE* in one patient from our cases (FA10). This alteration corresponded to a homozygous missense variant, which has been previously reported in association with FA.

*FANCE* is involved in the recruitment of *FANCD2* to sites of DNA damage, acting as a critical mediator within the FA/BRCA pathway. Variants affecting *FANCE* function may impair this recruitment process, leading to defective activation of downstream DNA repair mechanisms and contributing to the genomic instability observed in FA. As shown in Figure 2, the *FANCE* variant identified in our cohort is located within a region implicated in *FANCD2* recruitment, supporting its potential functional relevance.

## 3. Discussion

In our case series, short stature was the most frequent clinical feature, being present in all patients analyzed. Upper limb anomalies represented the second most common finding, followed by microphthalmia. Congenital abnormalities affecting the upper limbs, particularly those involving the thumb and radial ray, are considered classic features of FA and have been reported in approximately 43–82% of affected individuals in previous studies [2]. Although growth abnormalities are also well recognized in FA and have been described as one of the most common clinical manifestations in several reports [15], different clinical series emphasize radial anomalies as the most characteristic congenital finding of the disease [16]. In our patients, however, short stature was the most prevalent feature, followed by upper limb abnormalities, highlighting the phenotypic variability observed among different FA populations.

An additional observation in our study was the high frequency of microphthalmia, which was present in 71.4% of patients. This proportion appears to be higher than that reported in other cohorts, in which ocular anomalies such as microphthalmia have been described in approximately 11% of patients. However, no formal statistical comparison was made, and this difference should be interpreted with caution. These findings may reflect the clinical heterogeneity of FA and potential population-specific variation.

From a molecular perspective, *FANCA* was the most frequently altered gene in our study, accounting for 11 patients (78.6%), followed by *FANCC* in two patients (14.3%) and *FANCE* in one patient (7.1%). This distribution is largely consistent with previous reports describing *FANCA* as the most commonly mutated gene in FA, responsible for approximately 60–70% of cases worldwide [1]. Mutations in *FANCC* have been reported in a smaller proportion of patients, accounting for roughly 10% of cases, whereas *FANCE* variants are considerably rarer, representing approximately 1% of FA cases. Although each of these genes contributes differently to the overall mutational spectrum of the disease, *FANCA*, *FANCC*, and *FANCE* all encode proteins that are part of the FA core complex, which plays a central role in the activation of the FA/BRCA DNA repair pathway [17]. The predominance of *FANCA* variants observed in our study therefore aligns with the molecular distribution reported in other FA populations.

A further finding in our group of patients was the identification of two novel molecular alterations in *FANCA*, including the c.2782del variant and a large exonic deletion spanning exons 12 to 18. The c.2782del variant is predicted to result in a frameshift and premature truncation of *FANCA* protein, likely leading to loss of function, a well-established pathogenic mechanism in FA [18]. In addition, the identified exonic deletion further supports the role of structural variants as a relevant component of the mutational spectrum of *FANCA*. Together, these findings expand the known mutational landscape of *FANCA* and underscore the importance of integrating both sequencing-based approaches and copy number analysis to achieve a more comprehensive molecular characterization of FA patients.

Considering the three genes identified in our study, eight patients (57.1%) carried homozygous variants, whereas six (42.9%) presented compound heterozygous alterations. The distribution of homozygous and compound heterozygous variants may vary considerably among different Fanconi anemia cohorts and is often influenced by population-specific factors such as consanguinity or regional founder effects. Previous studies have reported a predominance of homozygous variants in populations with higher rates of consanguineous parents [19], whereas compound heterozygous variants are more frequently observed in genetically heterogeneous populations [20]. The relatively balanced distribution observed in our group of patients may therefore reflect the genetic heterogeneity of the Mexican population, in which both inheritance patterns are commonly encountered.

An additional observation in our study relates to the clinical presentation associated with different FA genes. The two patients carrying *FANCC* variants appeared to exhibit a more noticeable clinical phenotype compared with individuals harboring *FANCA* or *FANCE* mutations, presenting a greater number of clinical features according to the VACTERL-H and PHENOS criteria. However, given the very small number of cases in this subgroup, no formal statistical analysis was performed, and this observation should be considered preliminary rather than conclusive.

This finding may suggest a potential genotype–phenotype relationship, with *FANCC* variants possibly associated with a more pronounced clinical phenotype, consistent with earlier reports indicating an earlier onset of bone marrow failure in patients harboring *FANCC* mutations [21]. Interestingly, previous studies have also highlighted that the severity of the clinical phenotype may vary between populations, even among patients carrying the same variant; for example, individuals from the Japanese population were reported to present fewer congenital abnormalities compared with Ashkenazi Jewish patients carrying the same *FANCC* mutation [22]. Together, these findings underscore the clinical heterogeneity of FA and the potential influence of both genetic and population-specific factors. Nevertheless, larger cohorts in the Mexican population will be necessary to determine whether *FANCC* variants are consistently associated with a more pronounced clinical presentation.

A further notable finding was the recurrence of the *FANCA*:c.3931_3932del variant, which was identified in six patients, three of whom were siblings (FA5, FA6, and FA7) with a history of consanguinity. Interestingly, all individuals carrying this variant originated from Michoacan, Mexico, suggesting possible regional enrichment within our study group. However, this variant has also been reported in other populations, including Japanese and Turkish cohorts, indicating that it may represent a recurrent pathogenic variant rather than a population-specific founder mutation [23,24].

Previous studies have demonstrated the presence of founder variants in certain populations, such as the *FANCG* mutation reported in patients from Oaxaca [25], and a recurrent *FANCC* variant has been identified among the Mennonite population in Tamaulipas [26]. These examples highlight how historical migration patterns and population structure may influence the mutational spectrum of FA across different regions. Nevertheless, further genetic and population-based studies, including haplotype analysis, will be necessary to determine whether *FANCA*:c.3931_3932del shows evidence of regional aggregation or shared ancestry in patients from Michoacán.

Importantly, our study contributes to the clinical and molecular characterization of Fanconi anemia in a Mexican patient population, which remains underrepresented in the literature. The integration of phenotypic and genomic data in this group provides additional insight into the variability of disease presentation and the distribution of pathogenic variants in this population.

A limitation of this study is the lack of systematic orthogonal validation (e.g., Sanger sequencing) of the identified variants, as well as the absence of functional validation and haplotype analysis. Although the high-sequencing-quality metrics obtained and their consistency with previously reported variants support the reliability of our findings, further studies will be necessary to confirm the biological impact and population-level significance of the variants identified.

An additional limitation of this study is the relatively small sample size and the potential for selection bias. Patients were recruited based on clinical suspicion of Fanconi anemia, primarily in the context of bone marrow failure and associated phenotypic features, which may result in the overrepresentation of more severe clinical presentations. Furthermore, only patients with complete clinical and molecular data were included in the final analysis, which may limit the generalizability of our findings to the broader FA population. In addition, due to the limited sample size, no formal statistical comparisons were made, and, therefore, the findings should be interpreted as descriptive, for the generation of hypotheses.

## 4. Materials and Methods

### 4.1. Fanconi Anemia Patients

Patients were referred to our hospital between 2017 and 2025 for clinical evaluation due to cytopenias and suspicion of inherited bone marrow failure syndromes, particularly Fanconi anemia. Most patients were initially evaluated in the context of bone marrow failure (e.g., aplastic anemia) and associated phenotypic features suggestive of FA. Clinical suspicion was further supported by chromosomal instability testing. For each patient, pedigree, parental consanguinity, sex, age, somatometric data, and dysmorphic findings were collected.

### 4.2. Cytogenetic Analysis and Chromosome Breakage Test

From PB, conventional karyotyping was performed as described below; PB was cultured for 72 h at in Gibco RPMI 1640 medium (Gibco™, Thermo Fisher Scientific, Inc., Grand Island, NY, USA) and stimulated/supplemented with glutamine, phytohemagglutinin, fetal bovine serum, and antibiotic/antimycotic 100X (10,000 units/mL of penicillin, 10,000 μg/mL of streptomycin, and 25 μg/mL of Amphotericin B) (Gibco™, by Thermo Fisher Scientific Inc., Grand Island, NY, USA) at 37 °C with 5% CO_2_ incubation. Chromosome Giemsa–Trypsin–Wright staining was performed according to standardized protocol [27]. Chromosomes were karyotyped and analyzed following the International System for Human Cytogenomics Nomenclature [28].

For chromosome breakage assay, the PB cultures were treated with 0.1μg/ml diepoxybutane (Sigma Aldrich; Merck KGaA, St. Louis, MO, USA), and, for samples processed prior to 2022, they were treated with 50 ng/ml of Mitomicycin C (Sigma Aldrich; Merck KGaA, St. Louis, MO, USA) for 72 h with same protocol for conventional karyotyping. Simultaneously, PB cultures from healthy individuals were collected and analyzed as negative control, the procedure to be performed was explained to them as indicated in the informed consent form, and once they consented to participate, the PB sample was taken. A total of 100 independent metaphases were evaluated on the frequency of chromosome structural abnormalities per cell (chromatid gap, chromosome gap, dicentrics, rings, fragments, radial figures, and complex figures). The frequency of chromosomal abnormalities per cell and percentage of abnormality found were calculated.

### 4.3. Genomic Nucleic Acid Extraction

Genomic DNA was isolated from PB using QIAamp^®^ DNA Blood Mini kit (Qiagen GmbH, Germantown, MD, USA) according to the manufacturer’s instructions. Genomic isolated DNA integrity was analyzed by electrophoresis on 1% agarose gel stained with GelRed (Biotium, Fremont, CA, USA; Cat. No. 41003). DNA concentration was determined using spectrophotometry, with the absorbance (A) at 260 nm (A260) measured; genomic DNA “purity” was assessed by reading the A230 and A280, resulting in values of ~1.8 and 2.1 for A260/A280 and A260/A230 ratio, according to the results obtained with the NanoDrop™ One (NanoDrop Technologies; Thermo Fisher Scientific, Inc., Grand Island, NY, USA). Genomic DNA concentration from PB was analyzed by Qubit™ 4 Fluorometer Cat. Q33238 fluorometer (Invitrogen by Thermo Fisher Scientific, Inc., Grand Island, NY, USA), using Qubit™ dsDNA HS Assay Kit Cat. Q32851 (Invitrogen by Thermo Fisher Scientific, Grand Island, NY, USA) with a minimal concentration of 25 ng/μL.

### 4.4. MLPA Copy Number Variation Analysis

Based on genomic DNA from PB sample, MLPA probemixes were as follows: P031 *FANCA* Mix 1 (odd exons of *FANCA*), P032 *FANCA* Mix 2 (paired exons of *FANCA*), P113 *FANCB* Mix, P002 *BRCA2* Mix (*FANCD1*), and P057 *PALB2* Mix (*FANCD2*) (SALSA^®^ MLPA^®^ probemix by ©MRC HOLLAND, Amsterdam, The Netherlands). All individuals underwent MLPA targeting *FANCA* in order to detect potential copy number variations. When *FANCA* MLPA results were inconclusive, additional MLPA assays targeting *FANCD1*, *FANCD2*, and *FANCB* were performed to further evaluate possible CNVs. Fragment analysis was conducted using the Applied Biosystems™ SeqStudio™ Genetic Analyzer manufactured by Thermo Fisher Scientific. Fragments were analyzed with MLPA analysis software Coffalyser.NET v. 250317.1029 by ©MRC Holland. Results were reported following the International System for Human Cytogenetics Nomenclature [28]. No systematic orthogonal validation (e.g., qPCR or Sanger sequencing) was performed, which is acknowledged as a limitation.

### 4.5. Genotyping by NGS

Genomic DNA analysis was performed by a commercial targeted sequencing panel designed for germline hereditary cancer-associated mutations (panel size: 403 kb, 113 genes (covering all exons), 125 SNPs (48 ID SNPs and 77 SNPs for polygenic risk score), 10,341 oligo probes, TSHC panel (TruSight hereditary cancer panel; Illumina, Inc., San Diego, CA, USA). This germline TSHC panel includes 19/23 of FA causal genes (*FANCA*, *FANCB*/*FAAP95*, *FANCC*, *FANCD1*/*BRCA2*, *FANCD2*, *FANCE*, *FANCF*, *FANCG*/*XRCC9*, *FANCI*, *FANCJ*/*BRIP1*, *FANCL*, *FANCM*, *FANCN*/*PALB2*, *FANCO*/*RAD51C*, *FANCP*/*SLX4*, *FANCQ/ERCC4*, *FANCR*/*RAD51*, *FANCS*/*BRCA1*, and *FANCU*/*XRCC2*). *FANCT/UBE2T*, *FANCV*/*REV7*, *FANCW*/*RFWD3*, and *FANCX*/*FAP100* were not included.

Libraries were prepared using Illumina DNA Prep with Enrichment library prep chemistry, which includes library preparation and enrichment processes. Library quality was evaluated by a fragment analyzer system, 2100 Bioanalyzer Instrument Cat. G2939B; electrophoretic measurements were made using high-sensitivity DNA chips and high-sensitivity DNA reagents (Agilent Technologies, Santa Clara, CA, USA). Libraries quality results were evaluated by 2100 Expert Software (Version B.02.11.SI811).

Libraries sequencing was performed using MiSeq system instrument by Illumina^®^. This methodology enables the detection of single-nucleotide variants (SNVs), insertions/deletions (In/dels), and copy number variants (CNVs) in a single assay. The FASTQ files obtained were evaluated using the BaseSpace Enrichment App v3.1.0 (Illumina, Inc.) and with FastQC [29]. Analysis was performed in Franklyn on the Genoox platform (https://franklin.genoox.com/clinical-db/home (accessed on 19 March 2026)), and results were reported following the recommendations of the Human Genome Variation Society (HGVS) Nomenclature 2016 based on the human assembly GRCh37 (also known as hg19) (https://www.ncbi.nlm.nih.gov/datasets/genome/GCF_000001405.13/ (accessed on 19 March 2026)).

Sequencing quality metrics included an average depth of 182× for all samples analyzed, covering 113 genes related to hereditary cancer predisposition (TSHC panel by Illumina), including all exonic regions and 20 bp of flanking intronic sequences, (which is designed to ensure 95% uniformity, provided that the minimum depth is 100×). Variant calling was restricted to regions with adequate coverage and base quality (Phred score > 30). CNV variants were confirmed using the MLPA method.

Variants were classified according to the American College of Medical Genetics and Genomics (ACMG) guidelines, incorporating evidence from population databases, previously reported variants, and in silico prediction tools. Variants classified as VUS were evaluated in the context of the patient’s clinical presentation, inheritance pattern, in silico predictions, and associated genetic findings. As part of the in silico assessment, functional prediction models, including whole-genome-based approaches, were also reviewed and provided additional evidence suggestive of potential pathogenicity. However, as these data are not sufficient for definitive classification under ACMG criteria, such variants were considered potentially contributory to the phenotype only when consistent with the overall clinical and molecular context.

## 5. Conclusions

Taken together, our findings contribute to the clinical and molecular characterization of Fanconi anemia in a Mexican patient population, which remains underrepresented in the literature. Clinically, short stature was the most frequent manifestation observed, followed by upper limb anomalies and microphthalmia, reflecting the phenotypic variability of the disorder. From a molecular perspective, *FANCA* was the most frequently altered gene, consistent with its well-established predominance in FA, while variants in *FANCC* and *FANCE* accounted for a smaller proportion of cases.

In addition, two previously unreported *FANCA* variants were identified, including a frameshift alteration (c.2782del) and a large deletion spanning exons 12 to 18. The recurrence of the *FANCA*:c.3931_3932del variant in multiple patients from the same geographic region suggests possible regional enrichment within this study group, although its presence in other populations indicates that it may represent a recurrent variant rather than a population-specific founder mutation.

Overall, these findings highlight the value of integrating detailed clinical evaluations with genomic analysis for the diagnosis of FA and provide a descriptive framework that may support future studies in larger and more diverse populations.

## Figures and Tables

**Table 1:** Clinical features of the study cohort assessed according to the VACTERL-H and PHENOS criteria.

	FA1	FA2	FA3	FA4	FA5	FA6	FA7	FA8	FA9	FA10	FA11	FA12	FA13	FA14		
Sex	F	M	M	M	M	F	F	M	M	M	F	M	F	F	n	%
Age at diagnosis	9	11	3	6	15	11	13	11	6	5	4	5	11	18		
V	-	-	-	-	-	-	-	-	-	-	-	-	-	-	0/14	(0%)
A	-	-	-	-	-	-	-	-	-	-	×	-	-	-	1/14	(7.1%)
C	-	-	-	-	-	-	-	-	-	-	×	-	-	-	1/14	(7.1%)
T	-	-	-	-	-	-	-	-	-	-	-	-	-	-	0/14	(0%)
E	-	-	-	-	-	-	-	-	-	-	-	-	-	-	0/14	(0%)
R	×	×	×	-	-	-	-	-	-	-	×	-	-	-	4/14	(28.6%)
L	×	×	×	×	×	×	×	×	-	×	×	-	-	×	11/14	(78.6%)
H	-	-	-	-	-	-	-	-	-	-	-	-	-	-	0/14	(0%)
P	×	×	×	×	-	-	-	-	×	×	×	-	-	×	8/14	(57.1%)
H	×	×	×	×	-	-	-	-	-	-	×	×	×	-	7/14	(50%)
E	×	×	×	×	×	×	×	-	×	×	×	-	-	-	10/14	(71.4%)
N	-	-	-	-	-	-	-	-	-	-	-	-	-	-	0/14	(0%)
O	-	-	-	-	-	-	-	-	-	-	-	-	-	-	0/14	(0%)
S	×	×	×	×	×	×	×	×	×	×	×	×	×	×	14/14	(100%)

Age at diagnosis expressed in years. F = female; M = male. VACTERL-H: V, vertebral anomalies; A, anal atresia; C, cardiac anomalies; T, tracheoesophageal fistula; E, Esophageal atresia; R, renal anomalies; L, limb abnormalities; H, hydrocephalus. PHENOS: P, skin pigmentation abnormalities; H, small head (microcephaly); E, small eyes (microphthalmia); N, nervous system anomalies; O, otologic anomalies (hearing loss); S, short stature.

**Table 2:** Molecular characterization of our Fanconi anemia patients.

Patient	Gene	Exon/Intron	DNA Change	Protein Change	Mutation Type	rs	ACMG Classification	Genotype
FA1	*FANCA*	36	c.3555G>A	p.Trp1185*	Nonsense	rs1185165443	P	Compound heterozygous
	*FANCA*	29	c.2782del	p.Thr928Leufs*61	Frameshift (small deletion)	Novel	P	
FA2	*FANCA*	39	c.3931_3932del	p.Ser1311*	Nonsense	rs1403231932	P	Homozygous
FA3	*FANCC*	14	c.1513G>A	p.Ala505Thr	Missense	rs780179187	VUS	Compound heterozygous
	*FANCC*	11	c.1024C>G	p.Pro342Ala	Missense	rs863224606	VUS	
FA4	*FANCA*	12–18	c.1039-?_1747+?del	p.Val339_Leu586del**^1^**	Frameshift (large deletion)	Novel	P	Homozygous
FA5	*FANCA*	39	c.3931_3932del	p.Ser1311*	Nonsense	rs1403231932	P	Homozygous
FA6	*FANCA*	39	c.3931_3932del	p.Ser1311*	Nonsense	rs1403231932	P	Homozygous
FA7	*FANCA*	39	c.3931_3932del	p.Ser1311*	Nonsense	rs1403231932	P	Homozygous
FA8	*FANCA*	29	c.2851C>T	p.Arg951Trp	Missense	rs755546887	P	Compound heterozygous
	*FANCA*	i24	c.2222+8C>T	Not Known	Splicing	rs745775730	VUS	
FA9	*FANCA*	39	c.3931_3932del	p.Ser1311*	Nonsense	rs1403231932	P	Compound heterozygous
	*FANCA*	38	c.3788_3790del	p.Phe1263del	Frameshift (small deletion)	rs397507553	P	
FA10	*FANCE*	5	c.1111C>T	p.Arg371Trp	Missense	rs775076977	P	Homozygous
FA11	*FANCC*	13	c.1288_1290del	p.Tyr430del	Frameshift (small deletion)	rs1210997135	LP	Homozygous
FA12	*FANCA*	11	c.987_990del	p.His330Alafs*4	Frameshift (small deletion)	rs772359099	P	Compound heterozygous
	*FANCA*	1–3	c.-42_315+?del	No protein	Frameshift (large deletion)	Reported [8]	P	
FA13	*FANCA*	39	c.3931_3932del	p.Ser1311*	Nonsense	rs1403231932	P	Compound heterozygous
	*FANCA*	18–28	c.1659-?_2810+?del	p.Gly554_Lys937del ^1^	Frameshift (large deletion)	Reported [9]	P	
FA14	*FANCA*	29	c.2851C>T	p.Arg951Trp	Missense	rs755546887	P	Homozygous

^1^ Protein consequences of exon-level deletions are predicted based on the reference transcript and should be interpreted as estimated effects. P: Pathogenic; LP: Likely Pathogenic; VUS: Variant of Uncertain Significance.

## Data Availability

The data generated in the present study may be requested from the corresponding author.

## Abbreviations

The following abbreviations are used in this manuscript:

CI	Chromosome Instability test
CNVs	Copy Number Variations
FA	Fanconi Anemia
In/dels	Insertion/Deletions
ICLs	Interstrand CrossLinks
MLPA	Multiplex Ligation-dependent Probe Amplification
NGS	Next-Generation Sequencing
PB	Peripheral Blood
SNVs	Single-Nucleotide Variations
TSHC	TruSight Hereditary Cancer Panel
